# Reciprocal regulation of LOXL2 and HIF1α drives the Warburg effect to support pancreatic cancer aggressiveness

**DOI:** 10.1038/s41419-021-04391-3

**Published:** 2021-11-26

**Authors:** Rongkun Li, Hengchao Li, Lili Zhu, Xiaoxin Zhang, Dejun Liu, Qing Li, Bo Ni, Lipeng Hu, Zhigang Zhang, Yanli Zhang, Xu Wang, Shu-Heng Jiang

**Affiliations:** 1 Institute of Oncology, Affiliated Hospital of Jiangsu University, Zhenjiang, 212001 China; 2 State Key Laboratory of Oncogenes and Related Genes, Shanghai Cancer Institute, Ren Ji Hospital, School of Medicine, Shanghai Jiao Tong University, Shanghai, 200240 China; 3 Department of Pancreatic surgery, Huashan Hospital, Fudan University, Shanghai, 200040 China; 4 Jiangsu Key Laboratory of Medical Science and Laboratory Medicine, School of Medicine, Jiangsu University, Zhenjiang, 212013 China; 5 Department of Biliary-Pancreatic Surgery, Ren Ji Hospital, School of Medicine, Shanghai Jiao Tong University, Shanghai, 200127 China; 6 Department of Gastrointestinal Surgery, Ren Ji Hospital, School of Medicine, Shanghai Jiao Tong University, Shanghai, 200127 China

**Keywords:** Pancreatic cancer, Cell growth

## Abstract

Hypoxic microenvironment is common in solid tumors, particularly in pancreatic ductal adenocarcinoma (PDAC). The Warburg effect is known to facilitate cancer aggressiveness and has long been linked to hypoxia, yet the underlying mechanism remains largely unknown. In this study, we identify that lysyl oxidase-like 2 (LOXL2) is a hypoxia-responsive gene and is essential for the Warburg effect in PDAC. LOXL2 stabilizes hypoxia-inducible factor 1α (HIF1α) from prolyl hydroxylase (PHD)-dependent hydroxylation via hydrogen peroxide generation, thereby facilitating the transcription of multiple glycolytic genes. Therefore, a positive feedback loop exists between LOXL2 and HIF1α that facilitates glycolytic metabolism under hypoxia. Moreover, LOXL2 couples the Warburg effect to tumor growth and metastasis in PDAC. Hijacking glycolysis largely compromises LOXL2-induced oncogenic activities. Collectively, our results identify a hitherto unknown hypoxia-LOXL2-HIF1α axis in regulating the Warburg effect and provide an intriguing drug target for PDAC therapy.

## Background

Pancreatic ductal adenocarcinoma (PDAC) is one of the most intractable and lethal cancers and has been the seventh leading cause of cancer-related deaths worldwide [1, 2]. Unfortunately, despite the improvements in diagnostic and therapeutic strategies for PDAC, the prognosis is still dismal reflected by a 5-year overall survival rate around of 8% [3]. Thus, there is a great need for further mechanistic understanding that triggers PDAC progression to be used for more effective therapeutic approaches for the treatment of PDAC.

Hypoxic microenvironment is a common feature of solid tumors and is particularly notable in PDAC due to poor blood flow caused by the desmoplastic reaction [4, 5]. To cope with hypoxia, cancer cells have developed numerous adaptive responses, during which hypoxia-inducible factor 1 (HIF1) plays central roles by activating a host of hypoxia-responsive genes [5, 6]. HIF1 is comprised of HIF1α and HIF1β, wherein HIF1α serves as the major regulatory subunit responsible for its transcriptional function [7]. Both the stability and activity of HIF1α are oxygen-dependently regulated. Under normoxic conditions, HIF1α is hydroxylated by oxygen-dependent prolyl hydroxylases (PHDs), which enable the tumor suppressor von Hippel-Lindau (VHL) to bind to and mark HIF1α for rapid degradation through the ubiquitin-proteasome pathway. Under hypoxic conditions, prolyl hydroxylation of HIF1α is blocked, leading to HIF1α stabilization and nuclear translocation [6, 8]. In the nucleus, HIF1α dimerizes with HIF1β and binds to the hypoxia response elements (HREs) in the promoter regions of target genes involved in a plethora of pathophysiological processes.

Under hypoxic conditions, cancer cells are required to meet their oxygen demand by reprogramming their metabolic pathways, such as switching glycolytic reprogramming from oxidative phosphorylation to glycolysis [9]. Cancer cells exhibit aberrant metabolism characterized by high glycolysis even with sufficient oxygen, a phenomenon known as aerobic glycolysis or the Warburg effect [10, 11]. Such metabolic reprogramming not only improves cancer cell adaption potential to fluctuating oxygen tension, but also provides cancer cells with a proliferation advantage by producing ATP as well as glycolytic intermediates for biosynthesis of nucleotides, lipids, and proteins [10]. Simultaneously, through this metabolic alteration, more than 90% of glucose is converted to lactate, which facilitates acidification of the tumor microenvironment to favor tumor invasion and metastasis [12, 13].

Lysyl oxidase-like 2 (LOXL2) is a member of the lysyl oxidase (LOX) family, which are copper- and quinone-dependent amine oxidases that promote the cross-linking of collagen and elastin, major components of desmoplastic stroma and fibrosis [14]. The LOX family is constituted by five members, LOX and lysyl oxidase-like 1-4 (LOXL1-4), all of which share a highly conserved enzymatic domain in C-terminus required for the oxidative deamination of peptidyl-lysine residues in substrates, generating an aldehyde group and a byproduct, hydrogen peroxide [15]. Accumulated studies have documented the roles of LOXL2 in tumorigenesis [16], however, the relationship between LOXL2 and cancer metabolism remains unclear.

Here, we described that LOXL2, induced by hypoxia, couples the Warburg effect to tumor progression in PDAC. Mechanistically, LOXL2 stabilizes HIF1α by inhibiting HIF1α hydroxylation dependent on its catalytic activity through a hydrogen peroxide-mediated mechanism and enhances the expression of HIF1α target genes, thereby promoting aerobic glycolysis and PDAC progression. The results strongly suggest that the LOXL2-HIF1α positive feedback loop might be a potential target for PDAC cancer therapy.

## Results

### Overexpressed LOXL2 predicts poor prognosis in PDAC

**Fig. 1:**
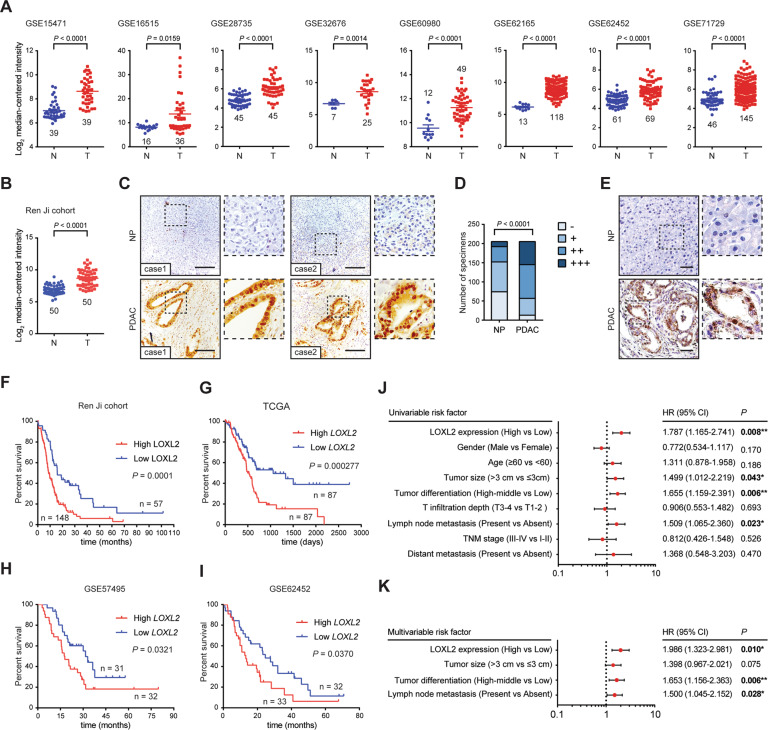
Expression pattern and prognostic value of LOXL2 in PDAC tissues. **A** *LOXL2* mRNA expression in PDAC tissues (T) and normal pancreas tissues (N) by analyzing multiple gene expression profiles from GEO database. **B** *LOXL2* mRNA expression in PDAC tissues (T) and normal pancreas tissues (N) from Ren Ji hospital published in GSE102238. **C** LOXL2 expression in the adjacent normal pancreas (NP) and PDAC tissues in tissue microarrays revealed by IHC staining and representative IHC images of LOXL2 in NP and PDAC tissues. Scale bar: 50 μm. **D** The percentage of tissue cores displaying no (−), weak (+), moderate (++), or strong (+++) IHC staining of LOXL2 in NP and PDAC tissues. **E** Representative IHC images of LOXL2 staining in NP and PDAC tissues from Kras^G12D/+^; Trp53^R172H/+^; Pdx1-Cre; (KPC) mice. Scale bar: 50 μm. **F** Comparison of overall survival of PDAC patients with different LOXL2 expression in Ren Ji cohort, **G** TCGA cohort, **H** GSE57495 cohort, and **I** GSE62452 cohort. Survival curves were calculated via the Kaplan-Meier method and analyzed by the log-rank test. **J** Univariate and **K** Multivariate analyses showing the association between LOXL2 expression and PDAC survival. CI Confidence interval, HR Hazard ratio. *p < 0.05, **p < 0.01.

To investigate the LOX family members in PDAC, we first analyzed their expression pattern in PDAC and normal pancreas tissues using Gene Expression Omnibus (GEO) datasets. As a result, *LOXL2* mRNA level in PDAC was significantly higher than that in normal pancreas as shown in Fig. 1A and Fig. S1A–D. So, we selected LOXL2 for further study in PDAC. Consistently, a significantly higher *LOXL2* expression was observed in Ren Ji cohort (GSE102238) (Fig. 1B). IHC analysis of LOXL2 in a tissue microarray revealed that high LOXL2 expression was observed in the majority of PDAC tissues but not in the non-tumor tissues (Fig. 1C, D). Moreover, LOXL2 expression was also remarkably upregulated in PDAC tissues of Kras^G12D/+^; Trp53^R172H/+^; Pdx1-Cre (KPC) mice as compared with normal pancreas (Fig. 1E). Of note, LOXL2 staining was mainly distributed in the nuclear and cytoplasm of PDAC cells (Fig. 1C, E).

To elucidate the clinical relevance, we analyzed the association between LOXL2 expression and the clinicopathologic features of PDAC patients. High LOXL2 expression was associated with more aggressive tumor behaviors, including larger tumor size, advanced T stage, and frequent distant metastasis (Table S1). Additionally, Kaplan-Meier curves showed that the overall survival times were shorter in patients with high LOXL2 than those with low LOXL2 expression (Fig. 1F), which was further supported by the survival analyses of The Cancer Genome Atlas (TCGA) and GEO (GSE57495 and GSE62452) cohorts (Fig. 1G–I). Univariate and multivariate analyses revealed that LOXL2 expression was an independent predictor for the overall survival in addition to tumor size, tumor differentiation, and lymph node metastasis (Fig. 1J, K). Taken together, these findings suggest that LOXL2 is commonly upregulated and may serve as a predictor of malignant progression in PDAC.

### Hypoxia induces LOXL2 expression of PDAC cells

**Fig. 2:**
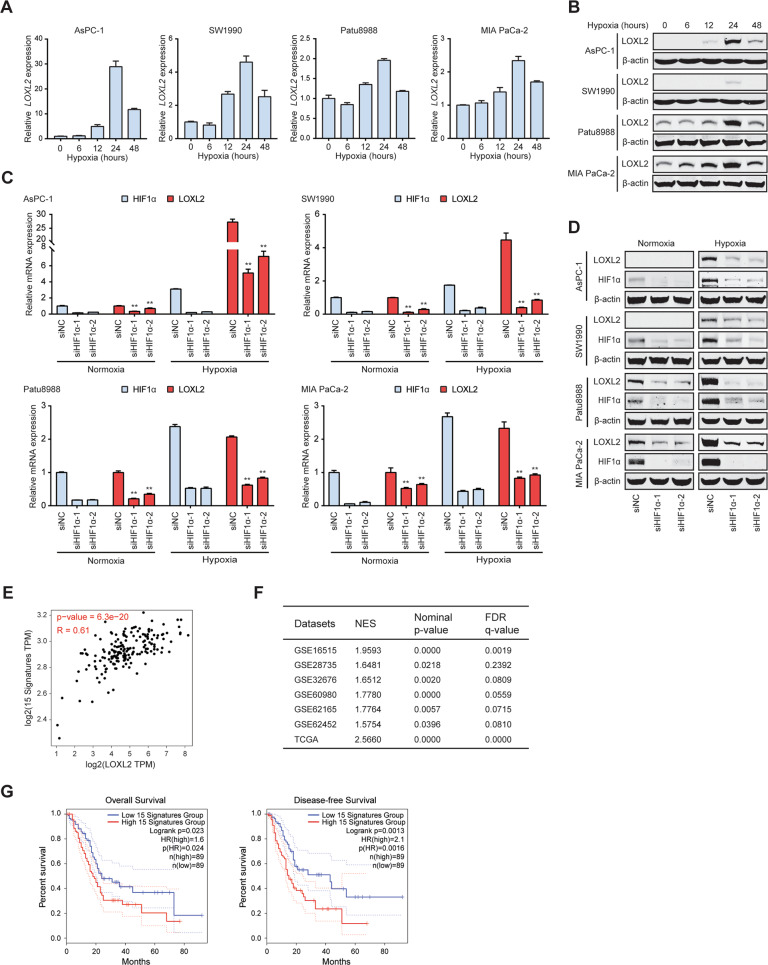
LOXL2 is induced by hypoxia in PDAC. **A** *LOXL2* mRNA expression in PDAC cells used for LOXL2 overexpression and knockdown exposed to hypoxia for 0–48 h examined by qRT-PCR. **B** LOXL2 protein expression in PDAC cells used for LOXL2 overexpression or knockdown exposed to hypoxia for 0–48 h examined by western blotting. **C**, **D** The expression of LOXL2 and HIF1α at mRNA (**C**) and protein (**D**) levels in the indicated cells transiently transfected with siRNAs targeting HIF1α exposed to normoxia or hypoxia for 24 h. **E** Correlation between LOXL2 expression and the 15-gene hypoxia signature in the PDAC samples of TCGA cohort. **F** Overall survival and disease-free survival analyses of PDAC patients with different hypoxia status in PDAC samples of TCGA cohort. **G** Positive correlation between *LOXL2* expression and gene set HALLMARK_HYPOXIA in PDAC samples analyzed by GSEA based on the data from GEO and TCGA databases. *p < 0.05, **p < 0.01.

To pursue the mechanism of LOXL2 dysregulation in PDAC, we tested whether LOXL2 expression could be induced by hypoxia. In four PDAC cells exposed to hypoxia for up to 48 h, LOXL2 was found to increase in hypoxia compared to normoxia at both mRNA (Fig. 2A) and protein (Fig. 2B) levels. Then, siRNAs targeting HIF1α were used to confirm whether LOXL2 expression induced by hypoxia is dependent on HIF1α in PDAC cells. As a result, HIF1α depletion drastically inhibited LOXL2 expression at both mRNA (Fig. 2C) and protein (Fig. 2D) levels during both normoxic and hypoxic conditions. Therefore, LOXL2 is induced by hypoxia in a HIF1α-dependent fashion. To determine whether LOXL2 and HIF1α are clinically pertinent, we analyzed the TCGA cohort for the expression of LOXL2 and a 15-gene hypoxia signature that reflects hypoxia status [17]. There was a significantly positive correlation between LOXL2 and the hypoxia signature (Fig. 2E), which was supported by the results of GSEA analyses using public GEO and TCGA databases that gene signatures related to hypoxia were significantly enriched in *LOXL2*-high samples compared with *LOXL2*-low samples (Fig. 2F). Furthermore, a high 15-gene hypoxia signature was associated with worse overall survival and disease-free survival in PDAC (Fig. 2G). These findings suggest that LOXL2 is a hypoxia-responsive gene aberrantly expressed in PDAC.

### LOXL2 promotes oncogenic progression in PDAC

**Fig. 3:**
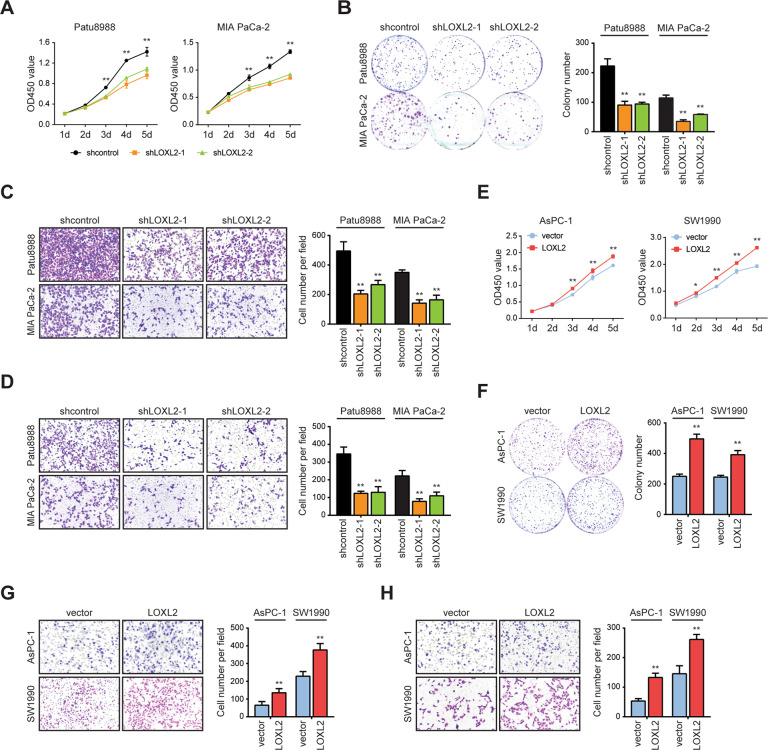
LOXL2 promotes PDAC cell proliferation, migration, and invasion in vitro. **A** CCK-8 assay of LOXL2 knockdown and control cells. **B** Colony formation assay of LOXL2 knockdown and control cells. **C** Transwell migration assay of LOXL2 knockdown and control cells. **D** Transwell invasion assay of LOXL2 knockdown and control cells. **E** CCK-8 assay of LOXL2-overexpressing and vector control cells. **F** Colony formation assay of LOXL2-overexpressing and vector control cells. **G** Transwell migration assay of LOXL2-overexpressing and vector control cells. **H** Transwell invasion assay of LOXL2-overexpressing and vector control cells. *p < 0.05, **p < 0.01.

**Fig. 4:**
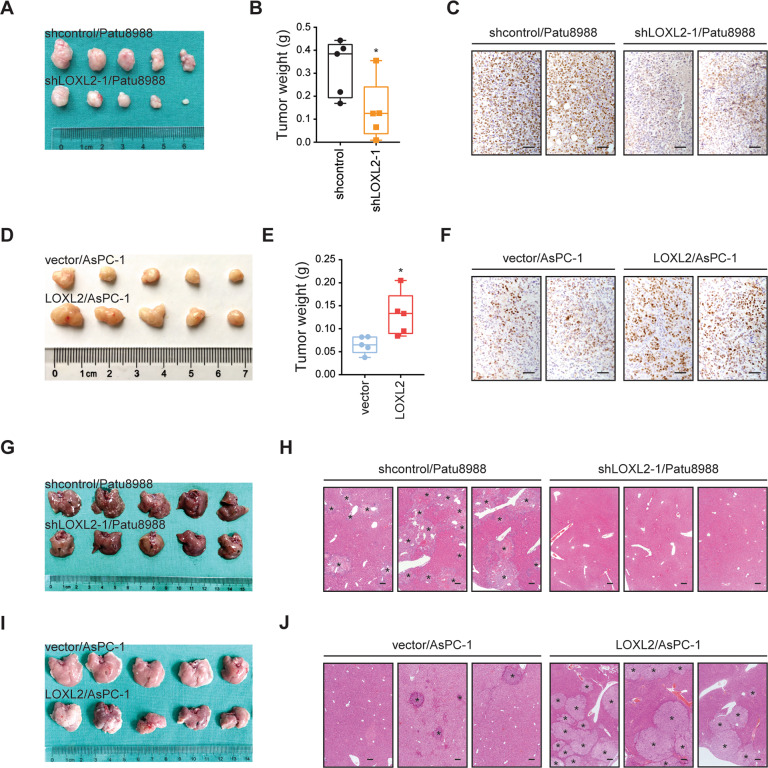
LOXL2 facilitates tumor growth and liver metastasis of PDAC in vivo. **A** Growth of subcutaneous xenografts from LOXL2 knockdown and control Patu8988 cells. **B** Weight of primary subcutaneous tumors of mice in (**A**). **C** Representative IHC images of PCNA staining in LOXL2 knockdown and control subcutaneous xenograft tissues. Scale bar: 50 μm. **D** Growth of subcutaneous xenografts from LOXL2-overexpressing and vector control AsPC-1 cells. **E** Weight of primary subcutaneous tumors of mice in (**D**). **F** Representative IHC images of PCNA staining in LOXL2-overexpressing and vector control subcutaneous xenograft tissues. Scale bar: 50 μm. (**G**) Liver metastasis of intrasplenic xenografts from LOXL2 knockdown and control Patu8988 cells. **H** Representative hematoxylin and eosin (H&E) images of liver metastasis in (**G**). The asterisks indicate liver metastasis lesions. Scale bar: 200 μm. **I** Liver metastasis of intrasplenic xenografts from LOXL2-overexpressing and vector control AsPC-1 cells. **J** Representative H&E images of liver metastasis in (**I**). The asterisks indicate liver metastasis lesions. Scale bar: 200 μm. *p < 0.05.

To determine the involvement of LOXL2 in the progression of PDAC, we first performed loss-of-function studies in two cell lines (Patu8988 and MIA PaCa-2) with higher LOXL2 expression (Fig. S2A, B). Two specific shRNAs against *LOXL2* led to marked downregulation of LOXL2 as revealed by qRT-PCR and western blotting (Fig. S2C, D). LOXL2 knockdown impaired PDAC cell proliferation and colony formation ability (Fig. 3A, B). Likewise, LOXL2 knockdown resulted in significantly reduced numbers of migrated cells (Fig. 3C) and invaded cells (Fig. 3D). Next, we established two LOXL2 overexpression cell models in AsPC-1 and SW1990 cells, which had lower endogenous LOXL2 expression (Fig. S2A, B and S2E, F). As expected, an opposite phenomenon was noticed in gain-of-function studies (Fig. 3E–H). To explore whether the in vitro findings could be recapitulated in vivo, we generated subcutaneous xenografts and an intrasplenic implantation mouse model. Compared to xenografts from the control group, LOXL2 knockdown robustly repressed tumor growth (Fig. 4A, B), which was further confirmed by IHC staining of the proliferation index PCNA (Fig. 4C). Furthermore, liver metastasis lesions were preferentially reduced with LOXL2 loss (Fig. 4G, H). Correspondingly, increased tumor growth (Fig. 4D–F) and liver metastasis (Fig. 4I, J) were obtained after LOXL2 overexpression. Altogether, these results suggest that LOXL2 confers growth and metastasis advantages to PDAC cells.

### LOXL2 enhances aerobic glycolysis of PDAC cells

**Fig. 5:**
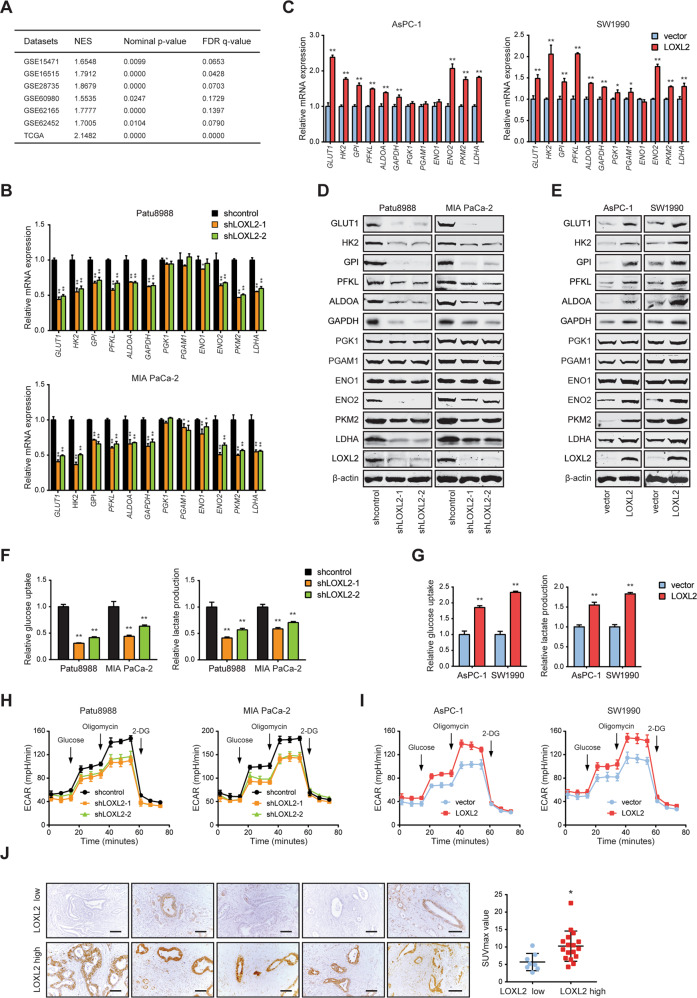
LOXL2 enhances aerobic glycolysis in PDAC cells. **A** Positive correlation between *LOXL2* expression and gene set HALLMARK_GLYCOLYSIS in PDAC samples analyzed by GSEA based on the data from GEO and TCGA databases. NES Normalized enrichment score, FDR False discovery rates. **B**, **C** Analysis of mRNA levels of glycolytic genes in LOXL2 knockdown and control cells (**B**) as well as in LOXL2-overexpressing and vector control cells (**C**) examined by qRT-PCR. **D**, **E** Analysis of protein levels of glycolytic genes in LOXL2 knockdown and control cells (**D**) as well as in LOXL2-overexpressing and vector control cells (**E**) examined by western blotting. **F** Glucose uptake and lactate production in LOXL2 knockdown and control cells. **G** Glucose uptake and lactate production in LOXL2-overexpressing and vector control cells. **H** Extracellular acid ratio (ECAR) in LOXL2 knockdown and control cells. **I** ECAR in LOXL2-overexpressing and vector control cells. **J** Representative IHC images of LOXL2 staining in PDAC tissues with the maximum standardized uptake value (SUVmax) (left panel) and comparison of the SUVmax between LOXL2-high and LOXL2-low patients (right panel). Scale bar: 50 μm. *p < 0.05, **p < 0.01.

To identify the mechanism by which LOXL2 promotes PDAC progression, we performed GSEA using public GEO and TCGA databases. As a result, gene signatures related to glycolysis were significantly enriched in *LOXL2*-high samples compared with *LOXL2*-low samples (Fig. 5A). Coincidentally, the medium culture of LOXL2 knockdown cells turned to be acidic much slower and later than that of control cells, especially under hypoxic conditions (Fig. S3A). These findings prompted us to speculate that LOXL2 might exert a role in regulating aerobic glycolysis in PDAC. The notion that LOXL2 regulates the Warburg metabolism of PDAC cells is supported by the results that (i) genetic manipulation of LOXL2 robustly affected the expression of glucose transporters and many glycolytic components at both mRNA and protein levels (Fig. 5B–E); (ii) LOXL2 knockdown attenuated glucose uptake, lactate production (Fig. 5F), and ECAR (an indicator for glycolysis) (Fig. 5H), while LOXL2 overexpression had the opposite effects (Fig. 5G, I). However, LOXL2 knockdown or overexpression slightly affected OCR (an indicator for oxidative phosphorylation) (Fig. S3B,C) and (iii) in a cohort of 25 PDAC patients who received preoperative ^18^F-fluorodeoxyglucose (^18^F-FDG) PET/CT, the maximum standardized uptake value (SUVmax), reflecting metabolic activity, was significantly higher in samples with high LOXL2 expression than that in samples with low LOXL2 expression (Fig. 5J).

Moreover, we grew cells in medium containing galactose instead of glucose, thereby reducing glycolytic flux and forcing the cells to rely on mitochondrial oxidative phosphorylation. Under this condition, LOXL2-overexpressing and vector control cells grew, migrated, and invaded at almost the same rate (Fig. S4A–D), indicating that inhibition of the Warburg effect greatly impeded the ability of LOXL2 to promote tumorigenesis. Likewise, we further treated the cells with 2-deoxy-D-glucose (2-DG), a glycolytic inhibitor, to block the glycolytic pathway. Notably, 2-DG also compromised the oncogenic activities of LOXL2 (Fig. S4E–H). Collectively, these data suggest that increased glycolysis is required for LOXL2 to facilitate PDAC progression and can be targeted by glycolysis inhibition.

### LOXL2 stabilizes HIF1α via inhibiting its PHD-dependent hydroxylation

**Fig. 6:**
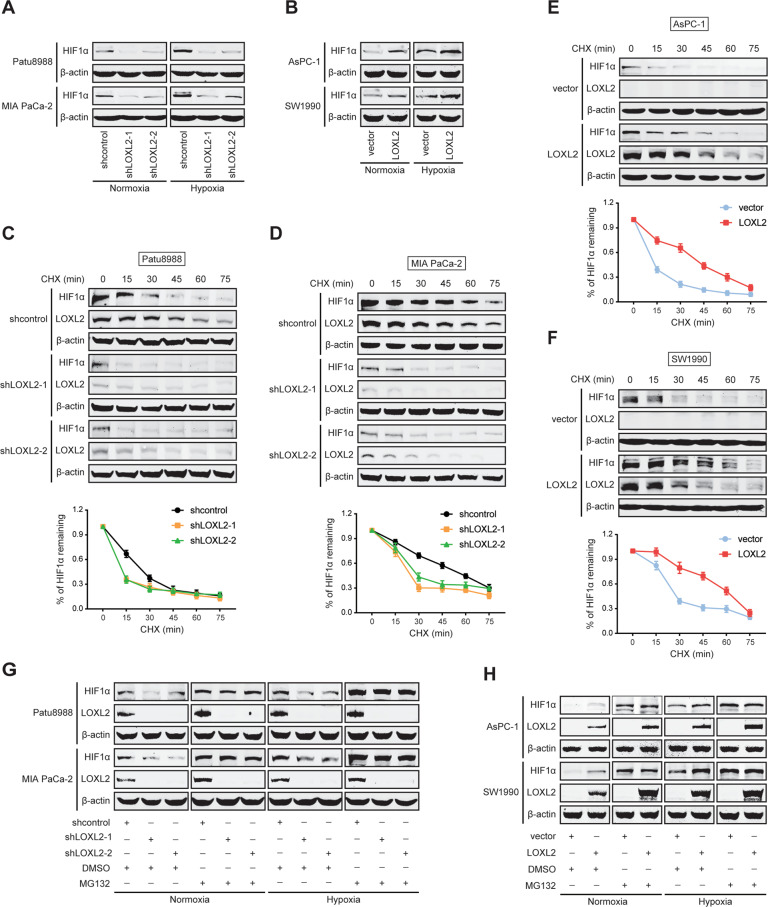
LOXL2 stabilizes HIF1α protein. **A** Western blotting showing HIF1α expression in LOXL2 knockdown and control cells under normoxic and hypoxic conditions. **B** Western blotting showing HIF1α expression in LOXL2-overexpressing and vector control cells under normoxic and hypoxic conditions. **C**, **D** HIF1α stability was examined by western blotting in LOXL2 knockdown and control cells. The cells were exposed to hypoxia for 6 h followed by incubation with 20 μg/ml cycloheximide (CHX) for the indicated times. **E**, **F** HIF1α stability was examined by western blotting in LOXL2-overexprssing and vector control cells. The cells were exposed to hypoxia for 6 h followed by incubation with 20 μg/ml CHX for the indicated times. **G** Western blotting showing HIF1α expression in LOXL2 knockdown and control cells treated with or without 10 μM MG132 for 6 h under normoxic and hypoxic conditions. **H** Western blotting showing HIF1α expression in LOXL2-overexpressing and vector control cells treated with or without 10 μM MG132 for 6 h under normoxic and hypoxic conditions.

HIF1α is a key transcriptional factor for the Warburg effect [7, 18]. Considering that multiple glycolytic enzymes regulated by LOXL2 are important transcriptional targets of HIF1α, we hypothesized that LOXL2 was involved in aerobic glycolysis via HIF1α. As expected, under both normoxic and hypoxic conditions, HIF1α protein levels were significantly reduced following LOXL2 knockdown (Fig. 6A), whereas increased following LOXL2 overexpression (Fig. 6B). *HIF1α* mRNA levels were not affected by LOXL2, suggesting that LOXL2-mediated regulation of HIF1α was translational or posttranslational (Fig. S5A, B), which was supported by the result from analysis of GSE35600 (Fig. S5C). Next, cells were exposed to hypoxia to stabilize HIF1α protein and then treated with cycloheximide (CHX) to arrest protein synthesis. Notably, LOXL2 knockdown shortened the half-life periods of HIF1α under hypoxic conditions (Fig. 6C, D), whereas HIF1α protein degraded at a slower rate after LOXL2 overexpression (Fig. 6E, F), indicating that LOXL2 stabilizes HIF1α. Then, MG132, a proteasome inhibitor, was utilized to prove that the effect of LOXL2 on HIF1α stability was dependent on proteasome-mediated degradation. MG132 treatment abolished altered HIF1α protein stabilization induced by LOXL2 knockdown (Fig. 6G) or overexpression (Fig. 6H). These results suggest that LOXL2 exerts a posttranslational regulatory effect on HIF1α protein stability, but not HIF1α protein translation. Next, we explored how LOXL2 regulates the stability of HIF1α protein. LOXL2 protein was observed intensively expressed in the nuclei of PDAC cells (Fig. S6A) and tissues (Fig. 1C and E), and HIF1α functions as a nuclear transcription factor, we therefore postulated a direct interaction between these two proteins because of their similar cellular localization. However, we failed to observe a physical interaction of LOXL2 with HIF1α (Fig. S6B, C), preliminarily ruling out the possibility that LOXL2 stabilizes HIF1α via direct interaction.

**Fig. 7:**
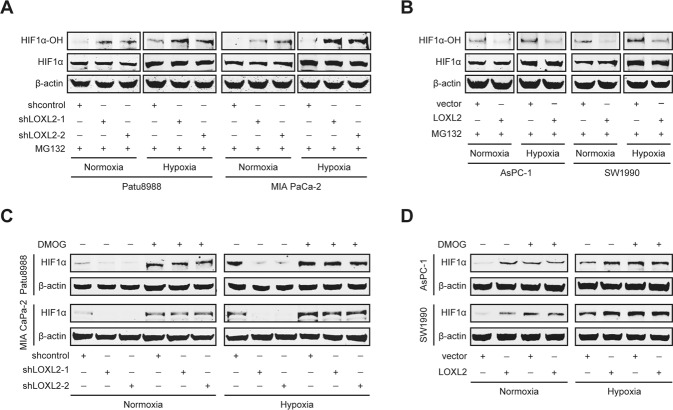
LOXL2 inhibits prolyl hydroxylase (PHD)-dependent hydroxylation of HIF1α. **A** Western blotting showing amounts of hydroxylated HIF1α (HIF1α-OH) and HIF1α in LOXL2 knockdown and control cells treated with 10 μM MG132 for 6 h under normoxic and hypoxic conditions. **B** Western blotting showing amounts of HIF1α-OH and HIF1α in LOXL2-overexpressing and vector control cells treated with 10 μM MG132 for 6 h under normoxic and hypoxic conditions. **C** Western blotting showing HIF1α expression in LOXL2 knockdown and control cells treated with or without 1 mM dimethyloxalylglycine (DMOG) for 24 h under normoxic and hypoxic conditions. **D** Western blotting showing HIF1α expression in LOXL2-overexpressing and vector control cells treated with or without 1 mM DMOG for 24 h under normoxic and hypoxic conditions.

Given that lots of oncogenes have been shown to stabilize HIF1α protein via inhibiting its hydroxylation [19–21], we tested whether LOXL2 stabilizes HIF1α by affecting its prolyl hydroxylation. To measure hydroxylated HIF1α levels, cells used for LOXL2 overexpression or knockdown were pretreated with MG132 to prevent hydroxylated HIF1α from being degraded. Less HIF1α but significantly more hydroxylated HIF1α was accumulated during MG132 treatment following LOXL2 knockdown (Fig. 7A), whereas the opposite effects were observed following LOXL2 overexpression (Fig. 7B), suggesting low HIF1α hydroxylation in the presence of LOXL2. HIF1α is hydroxylated on two proline residues (Pro402 and Pro564 in human HIF1α) by a family of oxygen-dependent prolyl hydroxylases (PHD1-4) [6, 8]. Therefore, we asked whether LOXL2 regulates HIF1α hydroxylation through PHD-dependent mechanism. It was validated by determination of the effect of treatment with dimethyloxalylglycine (DMOG), a potent PHD inhibitor, on HIF1α protein stability. If LOXL2 affects HIF1α stability by modulating PHD activity, DMOG treatment would overcome the effects of the manipulation of LOXL2 expression and produce equivalent levels of HIF1α. Indeed, equal levels of HIF1α were observed in response to DMOG treatment in the control and LOXL2 knockdown cells (Fig. 7C), as well as in the vector control and LOXL2-overexpressing cells (Fig. 7D). Overall, these results indicate that LOXL2 reduces PHD activity, and thus, impedes hydroxylation and enhances the stability of HIF1α protein.

### LOXL2 catalytic activity is indispensable for its functions in PDAC

**Fig. 8:**
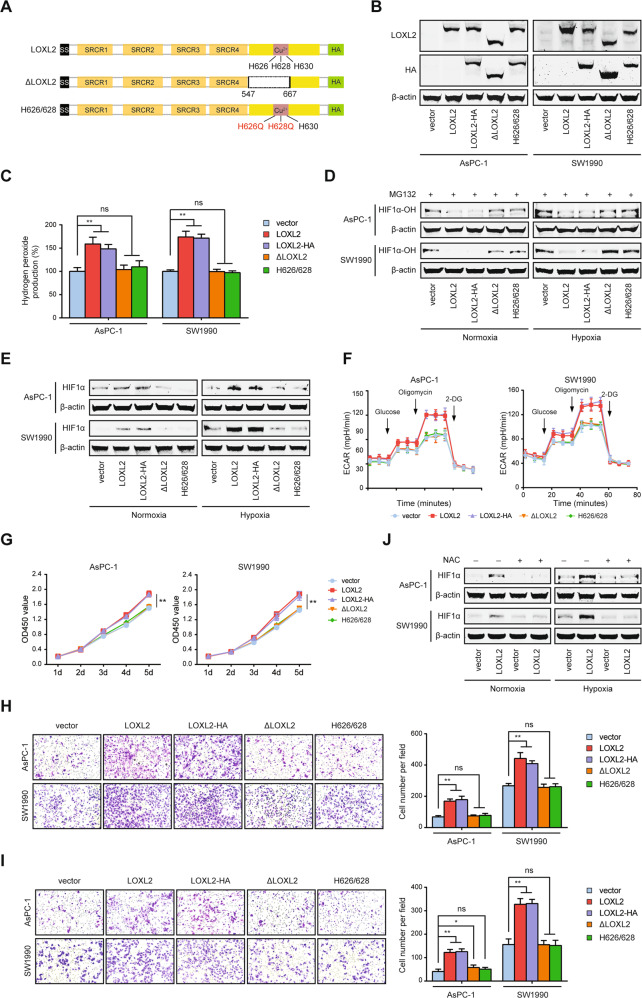
The catalytic activity is responsible for the oncogenic roles of LOXL2. **A** Schematic representation of wild-type LOXL2, deletion mutant (ΔLOXL2), and double point mutant (H626Q/H628Q) with the HA-tag. **B** Western blotting showing the indicated LOXL2 variants stably expressed in PDAC cells using anti-LOXL2 and anti-HA antibodies. **C** Intracellular hydrogen peroxide measured in PDAC cells stably transfected with the indicated LOXL2 variants. **D** Western blotting showing hydroxylated HIF1α (HIF1α-OH) expression in PDAC cells stably transfected with the indicated LOXL2 variants treated with 10 μM MG132 for 6 h under normoxic and hypoxic conditions. **E** Western blotting showing HIF1α expression in PDAC cells stably transfected with the indicated LOXL2 variants under normoxic and hypoxic conditions. **F** Extracellular acid ratio (ECAR) in PDAC cells stably transfected with the indicated LOXL2 variants. **G** CCK-8 assay of PDAC cells stably transfected with the indicated LOXL2 variants. **H**, **I** Transwell migration (**H**) and invasion (**I**) assays of PDAC cells stably transfected with the indicated LOXL2 variants. **J** Western blotting showing HIF1α expression in LOXL2-overexpressing and vector control cells treated with 10 mM N-acetylcysteine (NAC) for 24 h under normoxic and hypoxic conditions. *p < 0.05, **p < 0.01, ns No significance.

In addition to intracellular oxygen concentration, the activity of PHDs can also be regulated by several intracellular signals, including reactive oxygen species (ROS), which have been shown to inhibit the PHDs and stabilize HIF1α [22]. Hydrogen peroxide, an important member of ROS, is the byproduct of LOXL2 catalytic reaction. We, therefore, reasoned that HIF1α stabilization mediated by LOXL2 is dependent on hydrogen peroxide generation. To test this hypothesis, we generated two LOXL2 mutants affecting the conserved catalytic domain as described previously (Fig. 8A) [23, 24], which was applied to transfect AsPC-1 and SW1990 cells to clarify the implication of LOXL2 enzymatic activity on its action (Fig. 8B). LOXL2 enzymatic activity was subsequently determined by measurement of hydrogen peroxide production. As expected, LOXL2-overexpressing cells had an obvious increase in LOX catalytic activity compared with vector control cells, while both LOXL2 mutant cells had similar levels to vector control cells, confirming the absence of catalytic activity in LOXL2 mutants (Fig. 8C). Next, we tested whether or not LOXL2 depends on its catalytic activity to function as a positive regulator of PDAC. First, LOXL2 mutant cells had similar levels of HIF1α and hydroxylated HIF1α to control cells (Fig. 8D, E). Second, LOXL2 mutants failed to boost aerobic glycolysis (Fig. 8F) and the expression of glycolytic genes (Fig. S7). Third, LOXL2-mutant cells had similar proliferative, migratory, and invasive rates to those of control cells (Fig. 8G–I). Furthermore, we treated cells with the antioxidant N-acetylcysteine (NAC) to suppress hydrogen peroxide and hence block the downstream effects of LOXL2 mediated potentially by intracellular hydrogen peroxide. Indeed, the higher HIF1α level in LOXL2-overexpressing cells could be attenuated by NAC treatment (Fig. 8J). Accordingly, NAC treatment hijacked the oncogenic activities induced by LOXL2 overexpression (Fig. S8A–D). Collectively, the catalytic activity of LOXL2 and its byproduct, hydrogen peroxide, is required for its oncogenic roles in PDAC.

### LOXL2 stimulates aerobic glycolysis and tumor progression via HIF1α

To determine the involvement of HIF1α in LOXL2-mediated effects in PDAC, we depleted HIF1α in LOXL2-overexpressing cells (Fig. S9A, B). Depletion of HIF1α reversed enhanced aerobic glycolysis induced by LOXL2 overexpression (Fig. S9C). Moreover, depletion of HIF1α largely abolished the promoting effects of LOXL2 overexpression on cell proliferation, migration, and invasion (Fig. S9D–F). Together, these data indicate that HIF1α is indispensable for LOXL2-mediated tumor-promoting functions in PDAC cells.

## Discussion

**Fig. 9:**
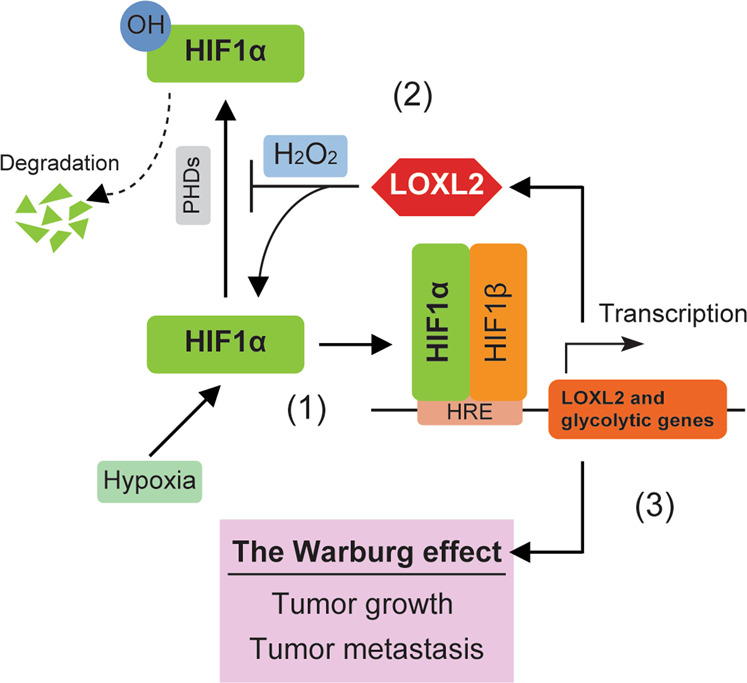
Model illustrating the mechanism regulated by LOXL2 in PDAC progression. **1** Hypoxia induces LOXL2 expression in a HIF1α-dependent fashion. **2** LOXL2 inhibits the hydroxylation of HIF1α to stabilize it via hydrogen peroxide (H_2_O_2_), the byproduct of LOXL2 catalytic activity, resulting in the expression of multiple glycolytic genes. **3** LOXL2 drives pancreatic cancer growth and metastatic progression dependent on the Warburg effect.

LOXL2 has been considered as a tumor promoter, how precisely it contributes to malignant phenotypes remains incompletely understood. Our present study establishes a forward feedback loop between LOXL2 and HIF1α as a positive regulator of aerobic glycolysis, which is vital for PDAC progression (Fig. 9). The LOXL2 induction following hypoxia is like to potentiate HIF1α signaling via blocking HIF1α hydroxylation dependent on its catalytic activity through a hydrogen peroxide-mediated mechanism. Given that hypoxia is a common feature of tumor microenvironment that drives tumor progression and that metabolic reprogramming is considered a new target for cancer therapy [25–27], our study may help to better understand the oncogenic potential of LOXL2 in the hypoxic microenvironment and metabolic reprogramming.

LOXL2 overexpression is observed in many human cancers, including gastric cancer [28], hepatocellular carcinoma (HCC) [29], breast cancer [30], and squamous cell carcinomas [31], and closely associated with their clinical-pathological characteristics and prognosis. Importantly, LOXL2 functions as an oncogene in various human cancers via diverse mechanisms [32–34]. For instance, LOXL2 facilitates intrahepatic metastasis by crosslinking extracellular matrix components in the liver to promote cytoskeletal remodeling [29]; LOXL2 interacts and cooperates with Snail to downregulate E-cadherin expression, thus inducing epithelial-to-mesenchymal transition (EMT) to drive carcinoma progression [33]. In pancreatic cancer, LOXL2 is involved in EMT induction, tumor progression, and gemcitabine resistance [35–37], but the underlying molecular mechanisms remain largely unknown. Consistent with LOXL2 promotion of tumor development and progression, LOXL2 knockdown and overexpression in human PDAC cell lines suggest oncogenic roles of LOXL2 in tumor growth and liver metastasis both in vitro and in vivo.

The Warburg effect is one of the most typical metabolic alterations in cancers. Emerging evidence supports the critical roles of the Warburg effect in promoting tumor initiation and progression [38]. Here, we demonstrated that LOXL2 could enhance aerobic glycolysis in PDAC cells, thus linking LOXL2 to tumor cell metabolic reprogramming. LOXL2 was found to upregulate the expression of multiple glycolytic genes, such as GLUT1, HK2, PKM2, and LDHA, leading to enhanced glycolytic activity. In addition, we showed that LOXL2-mediated glycolytic activation is required for its oncogenic roles in PDAC, suggesting a novel and critical role of LOXL2-mediated enhanced aerobic glycolysis in PDAC progression. With growing studies in tumor glucose metabolism, it has been realized that its increased activity is one of the major consequences of certain oncogenic drivers, among which HIF1α signaling represents the main pathway involved [39]. Intriguingly, LOXL2 indirectly enhances aerobic glycolysis in a HIF1α-dependent fashion. LOXL2 increases the levels of HIF1α protein and HIF1α depletion dramatically abrogates the promoting effects of LOXL2 overexpression on aerobic glycolysis as well as cell behaviors. Therefore, LOXL2-mediated HIF1α regulation represents a previously unknown mechanism that links aerobic glycolysis to PDAC progression.

HIF1α is mainly regulated at the level of protein stability [40]. HIF1α is maintained at low levels under normoxic conditions by collaboration between hydroxylation controlled by PHD1-4 and ubiquitination controlled by the VHL-containing E3 ubiquitin ligase complex [6, 8]. Here, LOXL2 was found to stabilize HIF1α via inhibiting PHD-dependent HIF1α hydroxylation upon the treatment of the PHD inhibitor DMOG. LOXL2 can exert its oncogenic roles via various mechanisms dependent or independent on its catalytic activity [16, 32, 41–43]. Hydrogen peroxide, as a byproduct of LOXL2 catalytic activity, belongs to ROS, which could inhibit the PHDs to stabilize HIF1α [22]. Our results indicated that the catalytic activity is indispensable for the oncogenic roles of LOXL2 in PDAC via generating LOXL2 mutants that lack the catalytic activity confirmed by measuring intracellular hydrogen peroxide levels. Moreover, LOXL2 functions as a PDCA promoter through a hydrogen peroxide-mediated mechanism. Taken together, LOXL2 enhances aerobic glycolysis via HIF1α stabilization dependent on its catalytic activity and thereby drives PDAC progression.

LOX family members have been reported to be induced by hypoxia and play critical roles in hypoxia-mediated tumor progression in several types of human cancers [44, 45]. In line with previous reports [46], LOXL2 expression in PDAC cells is induced by hypoxia in a HIF1α-dependent fashion. In view of the fact that LOXL2 stabilizes HIF1α, a feedforward loop is found between LOXL2 and HIF1α, which is confirmed by the observation in clinical PDAC tissues. A similar positive regulation loop between LOX and HIF1α was noticed in a previous study, which was proposed that the HIF1α-inducible LOX upregulates HIF1α protein synthesis via activating the PI3K-Akt signaling pathway dependent on LOX catalytic activity [46]. Distinct from this type of HIF1α regulation, our results uncover a HIF1α-regulatory mechanism in which LOXL2 upregulates HIF1α expression by stabilizing HIF1α from PHD-mediated hydroxylation, providing new molecular insights into how HIF1α is regulated by the LOX family.

LOXL2 has a complex role in cancers, which could be dependent or independent on its enzymatic activity, and at the same time, on its intracellular, extracellular, or intranuclear forms [47, 48]. Here, we propose the LOXL2-mediated stabilization of HIF1α in regulating PDAC progression and aerobic glycolysis dependent on its enzymatic activity through a hydrogen peroxide-mediated mechanism, but the involvement of other molecular mechanisms should not be ruled out, as LOXL2 is multi-functional. LOXL2 plays crucial intranuclear roles in the regulation of chromatin structure and gene transcription by mediating the oxidative deamination of lysine residues on target proteins, such as Snail1, H3K4me3, and TAF10 [49–51]. LOXL2 interacts with Snail1 to increase Snail1 stability [52], or to regulate heterochromatin transcription [49], thus inducing EMT. Unlike Snial1, despite intensive LOXL2 expression observed in the nuclei of PDAC tissues and cells, we did not detect the interaction between LOXL2 and HIF1α. Nevertheless, intranuclear roles of LOXL2 in PDAC warrant further study.

## Conclusions

Our study uncovers a previously unprecedented function of LOXL2 in PDAC progression via promoting aerobic glycolysis. Moreover, our results provide new mechanistic insights into the crucial roles of LOXL2 in HIF1α stabilization via inhibiting its hydroxylation dependent on its catalytic byproduct, hydrogen peroxide. The present suggests the feasibility of targeting the LOXL2-HIF1a feedback loop to inhibit aerobic glycolysis and to reverse the malignancy of PDAC, a cancer type for which existing therapeutic options are clinically insufficient.

## Materials and methods

### Clinical samples and IHC staining

Paraffin-embedded PDAC specimens were histopathologically diagnosed by the Department of Pathology, Ren Ji Hospital, School of Medicine, Shanghai Jiao Tong University. Two cohorts of specimens were used in this study: a tissue microarray containing 205 pathologist-certified and clinically annotated PDAC specimens and corresponding non-cancerous tissues and 25 PDAC tissues with available information of preoperative ^18^F-FDG PET/CT. All the patients did not receive chemotherapy, radiotherapy, or other adjuvant therapies prior to the surgery. Written informed consent was obtained from each patient. The use of these tissue samples was approved by the ethical review committee of the World Health Organization Collaborating Center for Research in Human Production (authorized by the Shanghai Municipal Government). IHC staining was performed using a two-step protocol as previously described [53]. For IHC analysis on xenograft tumors and pancreas tissues from KPC mice established by our laboratory [38], the specimens were fixed in 4% paraformaldehyde, embedded in paraffin and subjected to IHC staining. Primary antibodies used for IHC staining were LOXL2 (1:500, GeneTex, GTX105085) and PCNA (1:300, Cell Signaling Technology, #13110). Scoring was conducted based on both the ratio and intensity of the staining as previously reported [54].

### Cell culture and reagents

The human PDAC cell lines used in this study, including AsPC-1, BxPC-3, Capan-1, CFPAC-1, HPAC, MIA PaCa-2, PANC-1, Patu8988, and SW1990 were all preserved in Shanghai Cancer Institute, Ren Ji Hospital, School of Medicine, Shanghai Jiao Tong University. All PDAC cell lines were cultured in suggested medium (Hyclone, Logan, UT, USA) according to ATCC protocols, supplemented with 10% (v/v) fetal bovine serum (FBS, Hyclone, USA) and 100 Units/mL penicillin and 100 μg/mL streptomycin (Invitrogen, USA) at 37 °C in a humidified incubator with 5% CO_2_, except for MIA PaCa-2, which was cultured in medium supplemented with 10% (v/v) FBS, 2.5% (v/v) horse serum (Hyclone, USA), and 100 Units/mL penicillin and 100 μg/mL streptomycin. For hypoxic culture, PDAC cells were grown in a hypoxia incubator in an atmosphere consisting of 1% O_2_, 94% N_2_, and 5% CO_2_. The reagents used in the study were listed as follows: 2-DG (Sigma-Aldrich, D8375), CHX (Sigma-Aldrich, 239763-M), DMOG (Selleck, S7483), galactose (Sigma-Aldrich, G5388), MG132 (Selleck, S2619), and NAC (Sigma-Aldrich, A7250).

### Western blotting and co-immunoprecipitation

Standard western blotting was carried out using whole-cell protein lysates. Briefly, cultured cells were lysed with IP lysis buffer (Beyotime, Shanghai, China) supplemented with protease and phosphatase inhibitor cocktail (Bimake.cn, Houston, TX). Protein concentrations were determined using the Pierce BCA Protein Assay Kit (Thermo Fisher Scientific, USA) according to the manufacturer’s instructions. Cell lysates were analyzed by 5–10% sodium dodecyl sulfate-polyacrylamide gel electrophoresis (SDS-PAGE). Nitrocellulose (NC) membrane (Millipore, Danvers, MA) was used for gel transfer. After blocking with 5% non-fat milk, the membranes were hybridized overnight with primary antibodies: LOXL2 (1:1000, GeneTex, GTX105085), HIF1α (1:1000, Abcam, ab2185), Hydroxy HIF1α (1:1000, Cell Signaling Technology, #3434), GLUT1 (1:1000, Proteintech, 21829-1-AP), HK2 (1:5000, Proteintech, 22029-1-AP), GPI (1:1000, Proteintech, 15171-1-AP), PFKL (1:200, Santa Cruz, sc-393713), ALDOA (1:10000, Proteintech, 11217-1-AP), GAPDH (1:50000, Proteintech, 60004-1-Ig), PGK1 (1:1200, Proteintech, 17811-1-AP), PGAM1 (1:1000, Proteintech, 16126-1-AP), ENO1 (1:1000, Proteintech, 11204-1-AP), ENO2 (1:1000, Protrintech, 66150-1-Ig), PKM2 (1:1000, Cell Signaling Technology, #3198), LDHA (1:5000, Proteintech, 19987-1-AP), HA (1:1000, Millipore, 05-904), and β-actin (1:5000, MultiSciences, ab008). The next day, secondary antibodies conjugated with DyLight fluorescent dye (Thermo Fisher Scientific, USA) were incubated and the signal was detected using an Infrared Odyssey Imaging System (LI-COR Biosciences, Lincoln, NE, USA). For co-immunoprecipitation, cells were lysed as described in western blotting for 15 min on ice and supernatant was collected after centrifuging at 12,000 g for 15 min at 4 °C. Cell lysates were incubated with Pierce Anti-HA Magnetic Beads (Thermo Fisher Scientific, USA, #88836), anti-HIF1α (2 µg, Abcam, ab1), and anti-IgG (as a negative control, 2 µg, Abcam, ab200699) with rotation for 30 min at room temperature (RT), followed by incubation with Pierce Protein-A/G Magnetic Beads (Thermo Fisher Scientific, USA, #88803) for 15 min at RT. Immuno-complexes were washed three times with TBS-T or PBS-T and then resuspended in 1 × SDS-PAGE sample buffer for western blotting analysis.

### Quantitative real-time polymerase chain reaction (qRT-PCR)

Total RNA was extracted from cultured cells using the TRIzol total RNA isolation reagent (Takara Bio, Japan). Complementary DNA (cDNA) was prepared from 500 ng of total RNA using a prime Script RT reagent kit (Takara Bio, Japan) and subsequently subjected to qRT-PCR on ABI7500 instrument (Applied Biosystems) using 2 × SYBR Green qPCR Master Mix (Bimake, Shanghai, China) and specific primers listed in the Table S2. The expression of target genes was normalized to human *18sRNA*.

### Immunofluorescence analysis

All the PDAC cells used in this study were seeded on the confocal dishes and incubated overnight. Cells were fixed in 4% paraformaldehyde for 30 min, permeabilized with 0.2% Triton X-100 for 2.5 min, blocked in 1% bovine serum albumin (BSA) for 60 min, and incubated with primary antibodies against LOXL2 (1:200, GeneTex, GTX105085) for 60 min at RT, followed by Alexa Fluor 594-conjugated secondary antibodies (Molecular Probes, USA) for 30 min at RT. Nuclei were counterstained with 4’,6-diamidino-2-phenylindole (DAPI) (Vector Laboratories, Burlingame, CA, USA). Immunofluorescence signals were captured using laser confocal microscopy (Leica Microsystems AG).

### Transfection with siRNA

Small interfering RNAs (siRNAs) targeting HIF1α (siHIF1α) and non-targeting siRNAs (siNC) were synthesized by GenePharma Inc. (Shanghai, China) and transfected into PDAC cells according to the manufacturer’s instruction. Briefly, PDAC cells at 40 to 60% confluence were transfected with siRNAs using Lipofectamine 2000 (Invitrogen, Carlsbad, CA, USA) in optiMEM medium (Gibco, Invitrogen, Carlsbad, Calif, USA) for 6 h and then cultured in complete medium for another 48 h, followed by efficiency validation and subsequent function assays. The sequences targeting *HIF1α* gene were listed as follows: siHIF1α-1 (HIF1α-Homo-1168, 5’-3’: GAUGAAAGAAUUACCGAAUTT, 5’-3’ AUUCGGUAAUUCUUUCAUCTT), siHIF1α-2 (HIF1α-Homo-2090, 5’-3’: CUCCCUAUAUCCCAAUGGATT, 5’-3’ UCCAUUGGGAUAUAGGGAGTT).

### Lentivirus production and transfection

For overexpression, the plasmids expressing LOXL2, LOXL2-HA, and LOXL2 mutants were constructed by Shanghai Generay Biotech Co., Ltd. The cDNAs encoding full-length human LOXL2 (NM_002318), LOXL2-HA, and LOXL2 mutants were synthesized and inserted into pCDH-CMV-MCS-EF1-Puro vector (System Biosciences, Palo Alto, CA, USA). For knockdown, short hairpin RNA (shRNA) plasmids targeting LOXL2 were purchased from Shanghai Genechem Co., LTD. shRNA sequences targeting *LOXL2* gene were listed as follows: shLOXL2-1: 5’-GAAGGAGACATCCAGAAGAAT-3’; shLOXL2-2: 5’-GAGAGGACATACAATACCAAA-3’. 293T packaging cells were used to produce lentivirus, which was then transfected into target cell lines with 6 µg/ml polybrene for 24 h. Transfected cells used for overexpression or knockdown and their control cells were selected with 5 µg/ml puromycin for 2 weeks. The overexpression or knockdown efficiency of LOXL2 was assessed by qRT-PCR and western blotting.

### Hydrogen peroxide measurement

Intracellular hydrogen peroxide was measured using the Amplex Red Hydrogen Peroxide/Peroxidase Assay Kit (Invitrogen, Carlsbad, CA, USA) by a spectrophotometer according to the manufacturer’s instruction.

### Cell viability assay

Cell viability was determined using a Cell Counting Kit (CCK-8, Dojindo, Japan) according to the manufacturers’ instructions. Briefly, cells (1–2 × 10^3^ cells per well) were seeded into 96-well plates. The culture medium was removed and 100 μl of a 1:10 (v/v) dilution of CCK-8 in the medium was added to each well at the indicated time points. After incubated for 30–90 min in the cell incubator, the plates were measured at 450 nm using a multifunctional microplate reader (Bio-Rad Laboratories, Hercules, CA).

### Colony formation assay

Cells (1 × 10^3^ cells per plate) were seeded in 6 cm plates and allowed to grow for approximately 14 days. At the end of the experiments, colonies formed were washed with PBS twice, fixed with 4% paraformaldehyde for 30 min, and stained with 0.2% crystal violet for 1 h. Colonies larger than 100 μm in diameter for each plate were counted.

### Transwell migration and invasion assays

The capacity of cell migration and invasion was measured using 8-μm-pore Transwell chambers (Millipore, USA). For migration assay, 5 × 10^4^ cells in 200 μl serum-free medium were seeded into the upper chamber and allowed to migrate for 24 h. For invasion assay, 1 × 10^5^ cells in 200 μl serum-free medium were seeded into the upper chamber coated with Matrigel (BD Biosciences) and allowed to invade for 48 h. Meanwhile, 750 μl medium with 10% FBS in the lower chamber acted as a chemoattractant. 2-DG, galactose, and NAC were also added into the lower chamber. Subsequently, cells were fixed with 4% paraformaldehyde for 30 min and stained with 0.2% crystal violet for 1 h, followed by removing non-invading cells on the top surface of the chamber with cotton swabs. Finally, stained cells were counted in five randomly selected fields with 200 × magnification under a microscope to minimize the bias.

### Glucose consumption and lactate production assays

Cells were cultured to about 40% confluency in 6-well plates, and then incubated in fresh culture medium for additional 24 h. The culture medium was collected by centrifugation to remove the cells, and the levels of glucose (Catalog #: K676) and lactate (Catalog #: K607) were determined using kits from BioVision according to the manufacturer’s instructions. The values were normalized to the total protein amount determined using the Pierce BCA Protein Assay Kit (Thermo Fisher Scientific).

### Extracellular acidification rate (ECAR) and oxygen consumption rate (OCR) assays

The Seahorse XF96 Flux Analyzer (Seahorse Bioscience, Billerica, Massachusetts, USA) was used to measure ECAR and OCR of PDAC cells according to the manufacturer’s instructions. Approximately 1–2 × 10^5^ cells per well were seeded into an XF96-well plate and attached overnight. For the assessment of ECAR, cells were incubated with non-buffered RPMI 1640 under basal conditions followed by sequential injection of 10 mM glucose, 1 mM mitochondrial poison (oligomycin, Sigma-Aldrich, Saint Louis, Missouri, USA) and 80 mM glycolysis inhibitor (2-deoxyglucose, 2-DG, Sigma-Aldrich). OCR was assessed under basal conditions and after a sequential injection of 1 μM oligomycin, 1 μM fluoro-carbonyl cyanide phenylhydrazone (FCCP, Sigma-Aldrich, Saint Louis, Missouri, USA) and 2 mM antimycin A and rotenone (Sigma-Aldrich, Saint Louis, Missouri, USA). Both ECAR and OCR measurements were normalized by total protein content.

### Measurement of the half-life of HIF1α protein

Cells were seeded in 6-cm plates and exposed to hypoxia for 6 h to induce HIF1α protein. Then, CHX, a protein synthesis inhibitor, was used to treat the cells at a concentration of 20 μg/ml for 15, 30, 45, 60, and 75 min as indicated. The cell lysis was harvested followed by western blotting. The scale of the bands was analyzed by ImageJ software.

### Subcutaneous xenograft and intrasplenic inoculation models

In the subcutaneous model, 2 × 10^6^ cells (LOXL2-overexpressing AsPC-1 cells, LOXL2 knockdown Patu8988 cells, and their control cells) resuspended in 100 μl PBS were injected subcutaneously into the back of Balb/c nude mice (male, 5–6-week old, five mice per group). 5 weeks after subcutaneous inoculation, tumors were resected, weighed, embedded in paraffin, and subjected to IHC staining. In the intrasplenic inoculation model, 2 × 10^6^ cells (LOXL2-overexpressing AsPC-1 cells, LOXL2 knockdown Patu8988 cells, and their control cells) resuspended in 25 μl PBS were injected into the spleen of Balb/c nude mice (male, 5–6-week old, five mice per group). Mice harboring LOXL2 knockdown Patu8988 cells and their control cells were sacrificed 5 weeks after implantation, while mice harboring LOXL2-overexpressing AsPC-1 cells and their vector control cells were sacrificed 24 days after implantation, when mice harboring LOXL2-overexpressing AsPC-1 cells appeared with cachexia. The livers were dissected, fixed with 4% paraformaldehyde, embedded in paraffin, and subjected to hematoxylin and eosin (H&E) staining. Mice were manipulated and housed according to protocols approved by the East China Normal University Animal Care Commission. All mice received humane care according to the criteria outlined in the Guide for the Care and Use of Laboratory Animals prepared by the National Academy of Sciences and published by the National Institutes of Health.

### Bioinformatics analysis

The Gene Expression Omnibus (GEO) data and The Cancer Genome Atlas (TCGA) data referenced in the study are available in a public repository from the GEO website (https://www.ncbi.nlm.nih.gov/geo/) and the TCGA website (https://cancergenome.nih.gov/). *LOXL2* gene expression analysis was conducted using microarray gene expression data sets with the accession codes GSE15471, GSE16515, GSE28735, GSE32676, GSE60980, GSE62165, GSE62452, GSE71729, and GSE102238. Gene set enrichment analysis (GSEA, http://www.broadinstitute.org/gsea/index.jsp) was carried out based on the data from GEO and TCGA data sets. The pancreatic cancer samples in each data set were divided into two groups by the mean *LOXL2* expression level. GSEA was performed to compare the two groups within the Molecular Signatures Database of (h.all.v6.2.symbols.gmt). The gene sets showing a nominal *p* value less than 0.05 and false discovery rates (FDR) less than 0.25 were considered significantly enriched.

### Statistical analysis

Statistical analyses were performed using SPSS 19.0 for windows (IBM Corporation) and GraphPad Prism 7 software (San Diego, CA). The results were shown as mean ± SD and compared using a two-tailed, unpaired Student’s t-test or analysis of variance (ANOVA). Significant differences were accepted for *p* values of <0.05.

## Supplementary information

Supplementary information

## Data Availability

All data needed to evaluate the conclusions in the paper are present in the paper and/or the Supplementary Materials. Additional data related to this paper may be requested from the authors.
