# Supplementary material for: Reciprocal regulation of LOXL2 and HIF1α drives the Warburg effect to support pancreatic cancer aggressiveness
Source: Cell Death Dis. 2021 Nov 26;12(12):1106. doi: 10.1038/s41419-021-04391-3 (PMC8626482; doi:10.1038/s41419-021-04391-3)
Supplement: Supplementary file 1 — Supplementary information [file 41419_2021_4391_MOESM1_ESM.pdf]

# Supplementary Information

## Supplementary Figures

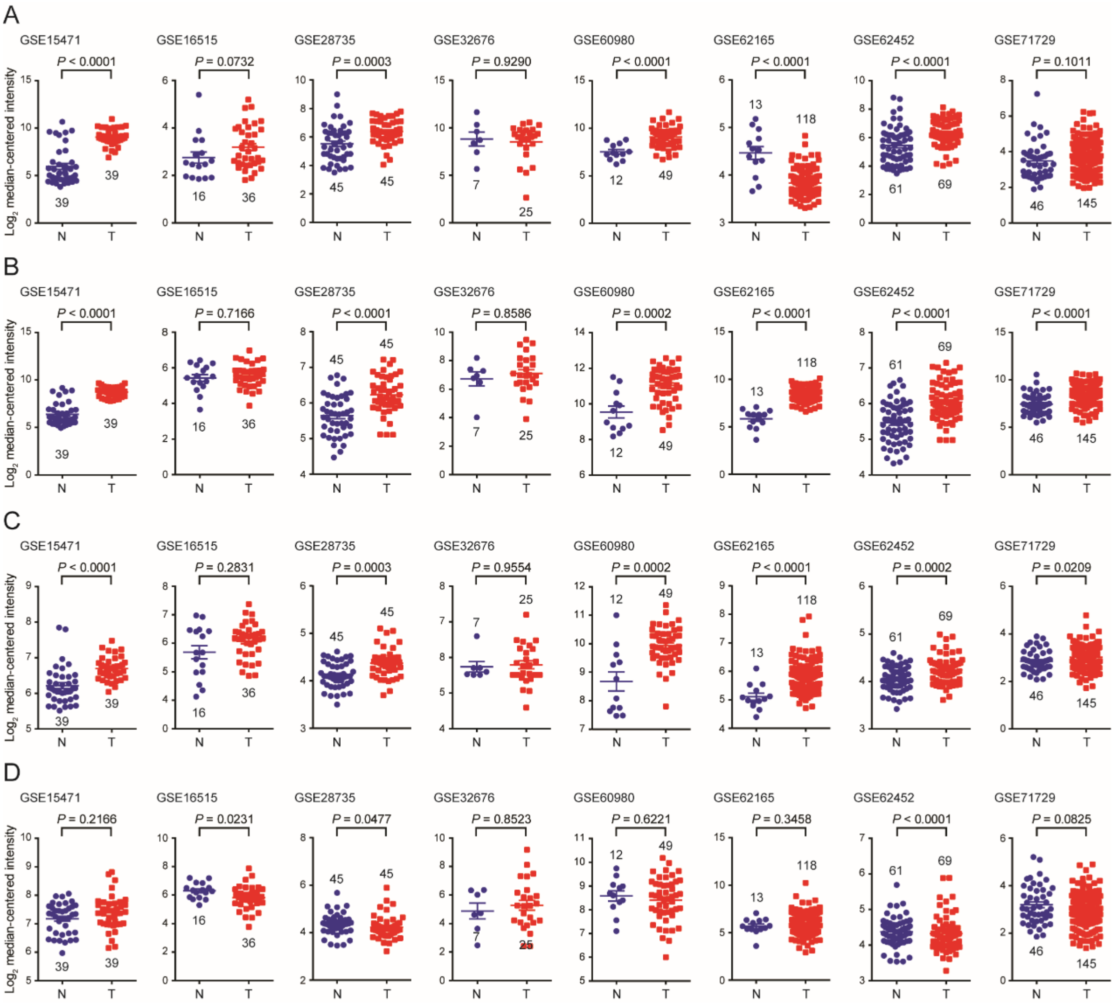


**Fig. S1.** The mRNA expression of *LOX* (A), *LOXL1* (B), *LOXL3* (C), and *LOXL4* (D) in PDAC tissues (T) and normal pancreas tissues (N) by analyzing multiple gene expression profiles from GEO database.

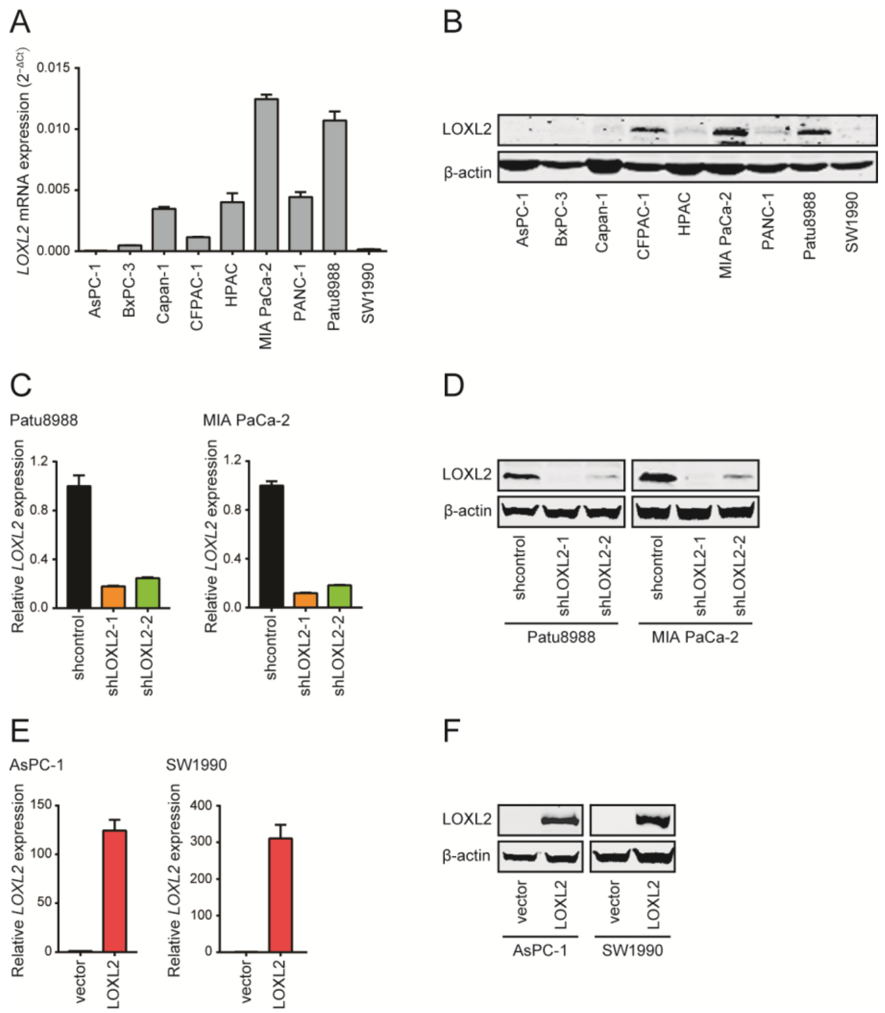


**Fig. S2. LOXL2 expression in PDAC cell lines and validation of LOXL2 knockdown and overexpression efficiency.** (**A**) *LOXL2* mRNA expression in PDAC cell lines examined by qRT-PCR. (**B**) LOXL2 protein expression in PDAC cell lines was examined by western blotting. (**C**) *LOXL2* mRNA expression in LOXL2 knockdown and control cells was confirmed by qRT-PCR. (**D**) LOXL2 protein expression in LOXL2 knockdown and control cells was confirmed by western blotting. (**E**) *LOXL2* mRNA expression in LOXL2-overexpressing and vector control cells was confirmed by qRT-PCR. (**F**) LOXL2 protein expression in LOXL2-overexpressing and vector control cells was confirmed by western blotting.

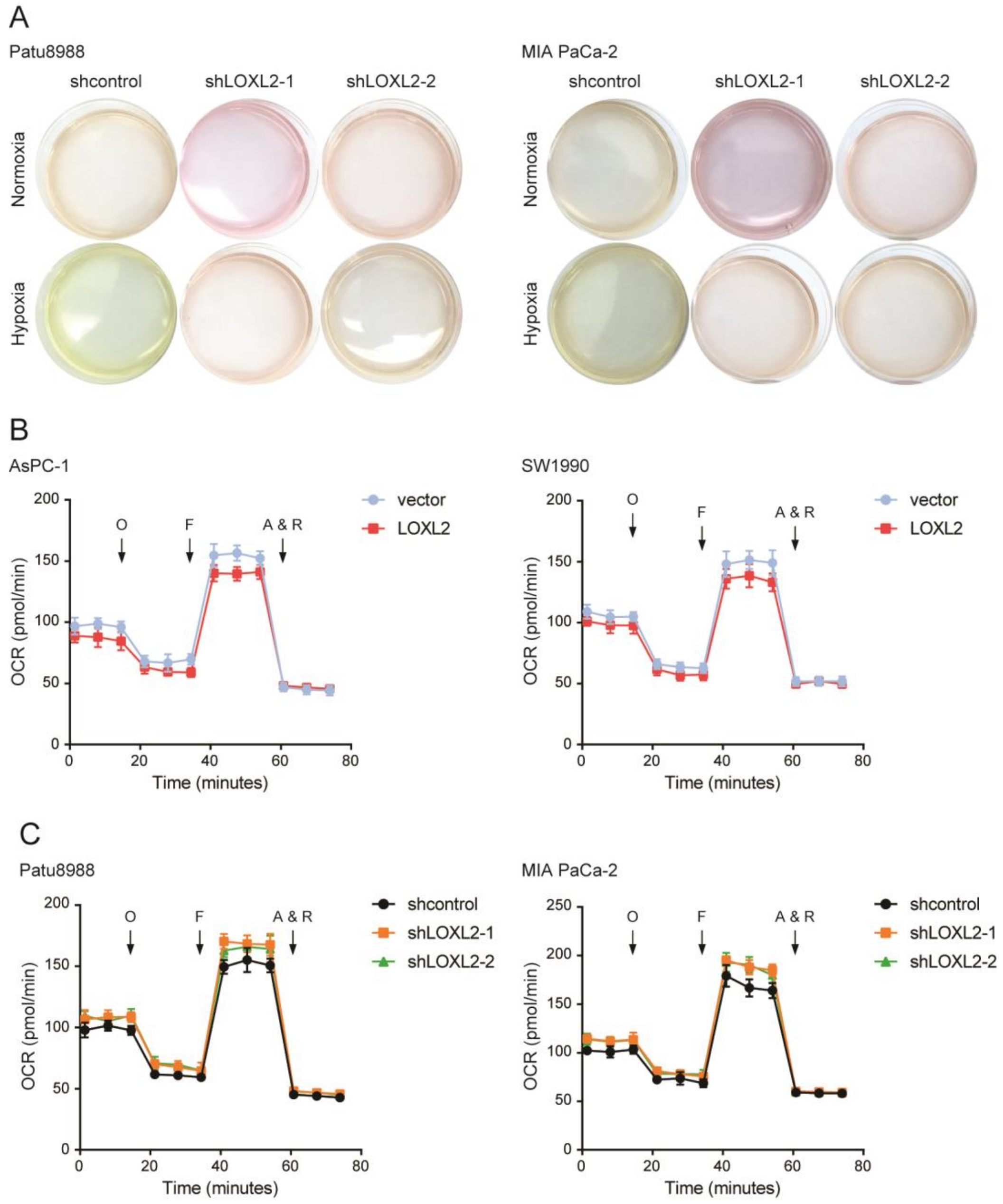


**Fig. S3. The effects of LOXL2 on aerobic glycolysis in PDAC cells.** (**A**) Cell culture dishes containing Patu8988 or MIA PaCa-2 cells with or without LOXL2 knockdown under normoxic or hypoxic conditions for 24 hours. (**B**) Oxygen consumption rate (OCR) in LOXL2 knockdown and control cells. (**C**) OCR in LOXL2-overexpressing and vector control cells. O, oligomycin; F, FCCP (carbonyl cyanide 4-[trifluoromethoxy] phenylhydrazone); A & R, antimycin A and rotenone.

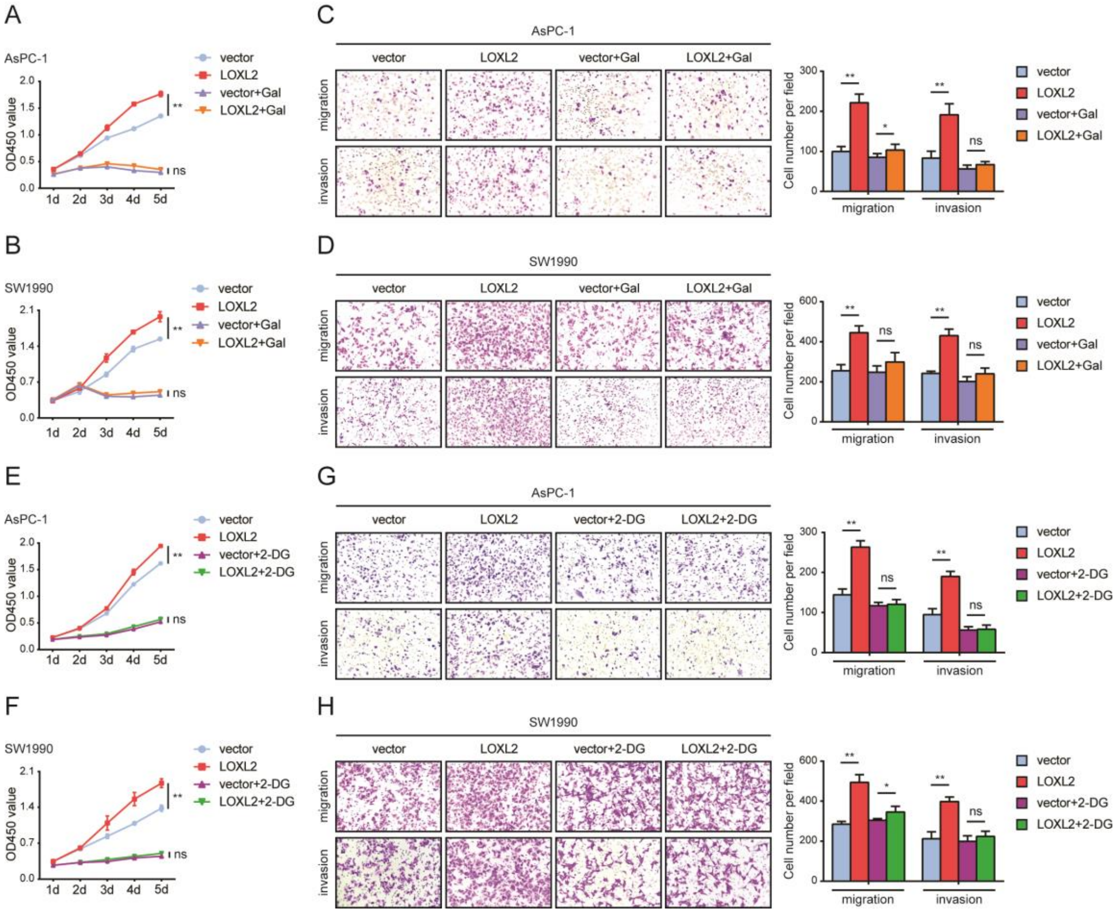


**Fig. S4. Aerobic glycolysis mediates the promoting effects of LOXL2 on PDAC cell proliferation, migration, and invasion.** (**A** and **B**) CCK-8 assay of LOXL2-overexpressing and vector control cells when cultured in medium containing galactose (Gal) but no glucose or normalized glucose. (**C** and **D**) Transwell migration and invasion assays of LOXL2-overexpressing and vector control cells when cultured in medium containing Gal but no glucose or normalized glucose. (**E** and **F**) CCK-8 assay of LOXL2-overexpressing and vector control cells when cultured in presence or absence of 2-deoxy-D-glucose (2-DG). (**G** and **H**) Transwell migration and invasion assays of LOXL2-overexpressing and vector control cells when cultured in presence or absence of 2-DG. *$p$ < 0.05, **$p$ < 0.01; ns, no significance.

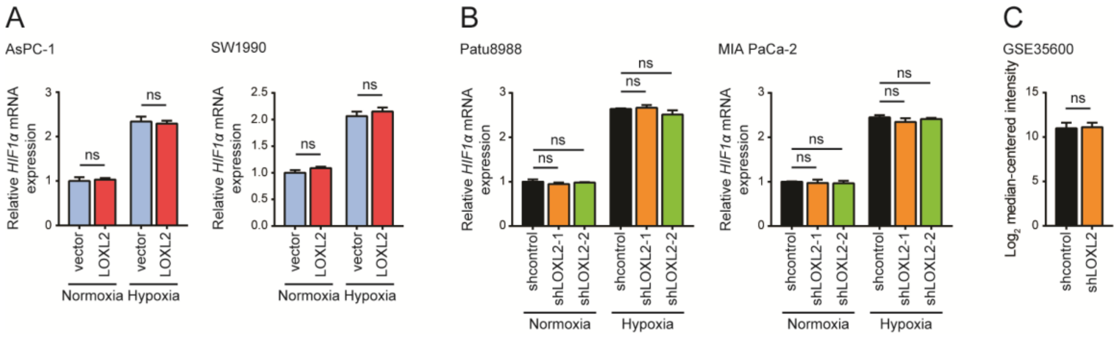


**Fig. S5. *HIF1α* mRNA expression after LOXL2 knockdown or overexpression in PADC cells.** (**A** and **B**) *HIF1α* mRNA expression after LOXL2 knockdown (A) or overexpression (B) under normoxic and hypoxic conditions examined by qRT-PCR. (**C**) *HIF1α* mRNA expression by analyzing the data from GSE35600. ns, no significance.

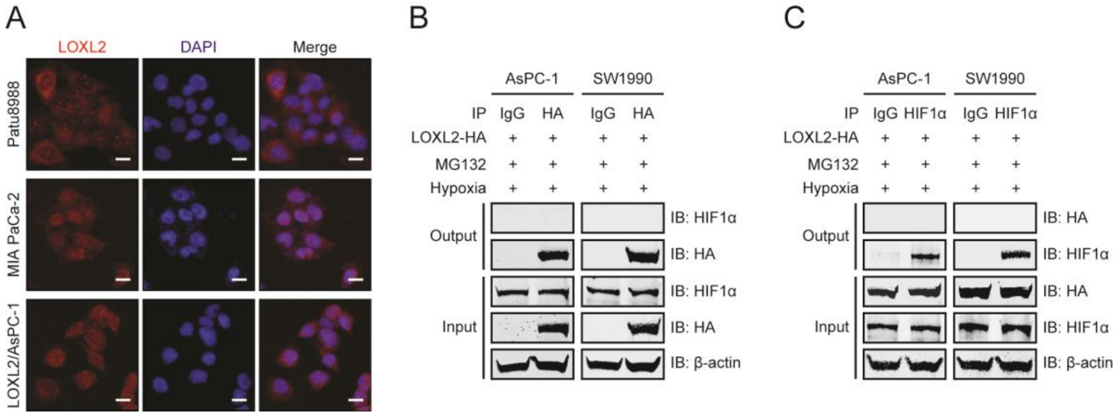


**Fig. S6. The interaction between LOXL2 and HIF1α in PDAC cells.** (**A**) Immunofluorescence images of endogenous LOXL2 in wide-type PDAC cells, Patu8988 and SW1990, and exogenous LOXL2 in LOXL2-overexpressing AsPC-1 (LOXL2/AsPC-1) cells. Scale bar: 20 μm. (**B**) LOXL2-HA overexpressing and vector control cells were incubated with 10 μM MG132 under hypoxic conditions for 6 hours. Exogenous LOXL2 was immunoprecipitated with anti-HA, and HIF1α was identified using anti-HIF1α. (**C**) LOXL2-HA overexpressing and vector control cells were incubated with 10 μM MG132 under hypoxic conditions for 6 hours. Endogenous HIF1α was immunoprecipitated with anti-HIF1α, and LOXL2 was identified using anti-HA.

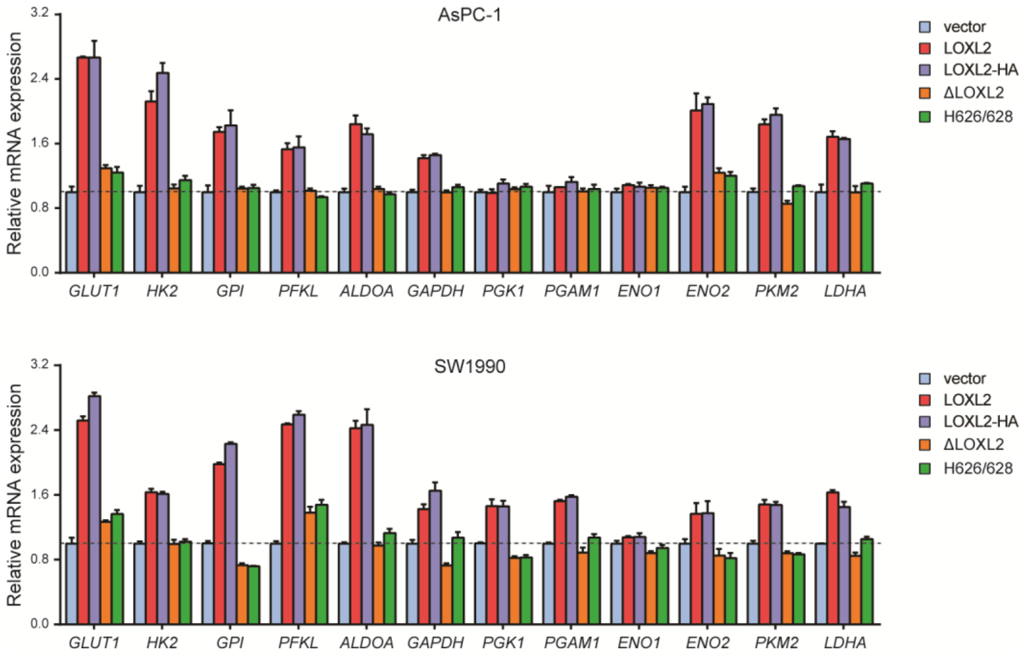


**Fig. S7. The expression of glycolytic genes at mRNA level in PDAC cells transfected with LOXL2 variants.** Analysis of mRNA levels of glycolytic genes in AsPC-1 and SW1990 cells stably transfected with LOXL2 variants as well as in LOXL2-overexpressing and vector control cells revealed by qRT-PCR.

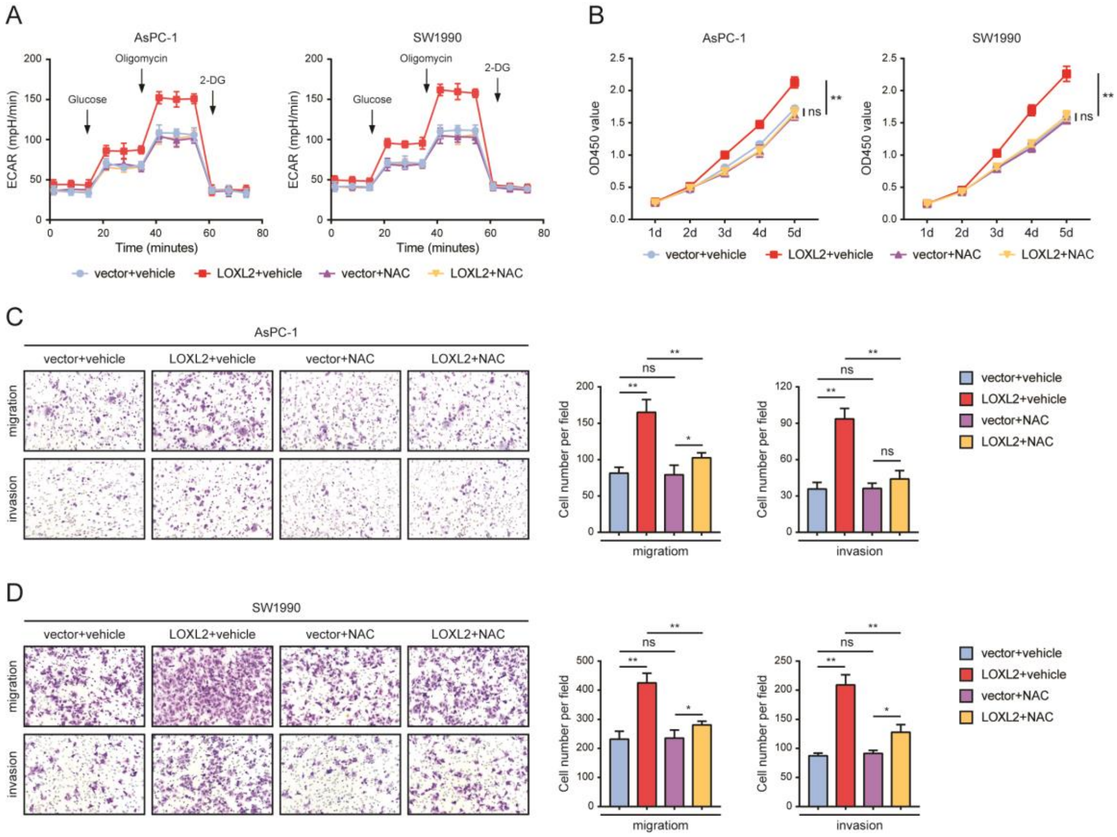


**Fig. S8. The effects of N-acetylcysteine (NAC) on aerobic glycolysis and PDAC progression mediated by LOXL2.** (**A**) Extracellular acid ratio (ECAR) in LOXL2-overexpressing and vector control cells treated with 10 mM NAC for 24 hours. (**B-D**) CCK-8 assay (B), Transwell migration assay (C), and Transwell invasion assay (D) of LOXL2-overexpressing and vector control cells treated with 1 mM NAC for 24 hours. *$p$ < 0.05, **$p$ < 0.01; ns, no significance.

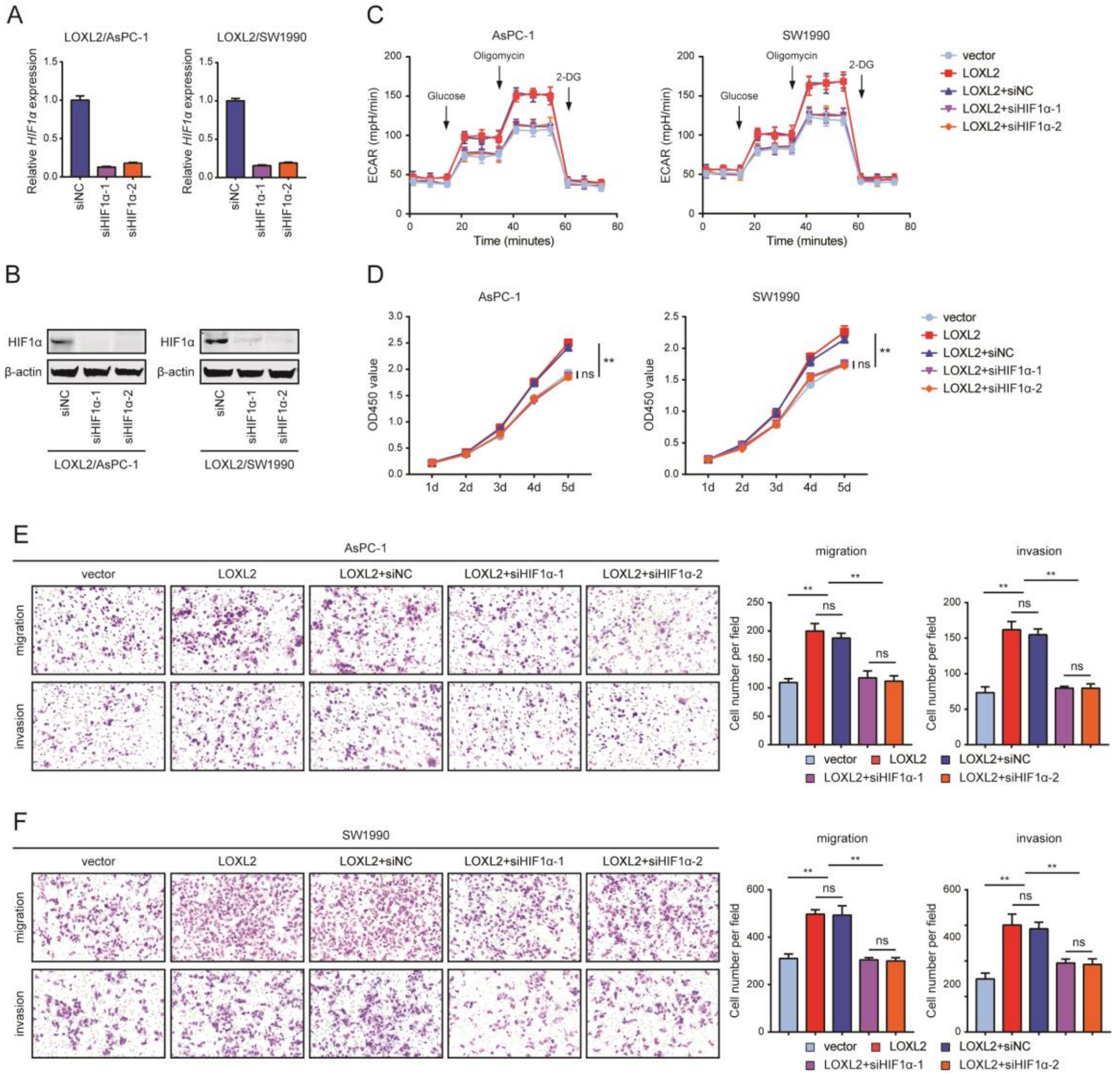


**Fig. S9. HIF1α mediates the promoting effects of LOXL2 on aerobic glycolysis and tumor progression.** (**A** and **B**) HIF1α protein expression in LOXL2-overexpressing cells transiently transfected with siRNAs targeting HIF1α confirmed by qRT-PCR (A) and western blotting (B). (**C**) Extracellular acid ratio (ECAR) in vector control cells and LOXL2-overexpressing cells transiently transfected with siRNAs targeting HIF1α. (**D-F**) CCK-8 assay (D), Transwell migration assay (E), and Transwell invasion assay (F) of vector control cells and LOXL2-overexpressing cells transiently transfected with siRNAs targeting HIF1α. $^{**}p < 0.01$; ns, no significance.

**Table S1. Correlations between LOXL2 expression and clinicopathologic parameters in patients with PDAC**

| Clinicopathological parameter | Total 205 | Expression of LOXL2 Low (n = 57, %) | Expression of LOXL2 High (n = 148, %) | *p* value |
|---|---|---|---|---|
| **Age (years)** | | | | |
| < 60 | 66 | 24 (36.4) | 42 (63.6) | 0.059 |
| ≥ 60 | 139 | 33 (23.7) | 106 (76.3) | |
| **Gender** | | | | |
| Male | 117 | 34 (29.1) | 83 (70.9) | 0.644 |
| Female | 88 | 23(26.1) | 65 (73.9) | |
| **Tumor size** | | | | |
| ≤ 2 cm | 27 | 14 (51.9) | 13 (48.1) | **0.003** |
| > 2 cm | 178 | 43 (24.2) | 135 (75.8) | |
| **T classification** | | | | |
| T1, 2 | 42 | 17 (40.5) | 25 (59.5) | **0.04** |
| T3, 4 | 163 | 40 (24.5) | 123 (75.5) | |
| **Lymph node metastasis** | | | | |
| Absent | 136 | 36 (26.5) | 100 (73.5) | 0.549 |
| Present | 69 | 21 (26.5) | 48 (73.5) | |
| **Distant metastasis** | | | | |
| Absent | 191 | 57 (29.8) | 134 (70.2) | **0.016** |
| Present | 14 | 0 (0) | 14 (100.0) | |

[a]The bold number represents the *p*-values with significant differences.

[b]*P* value was calculated by $\chi^2$ test or Fisher's exact test.

**Table S2. The primer sequences used in qRT-PCR analysis**

| Primer | Sequence (5'-3') |
|---|---|
| *18sRNA* Forward | TGCGAGTACTCAACACCAACA |
| *18sRNA* Reverse | GCATATCTTCGGCCCACA |
| *LOXL2* Forward | CCAGTGTGGTCTGCAGAGAG |
| *LOXL2* Reverse | CCTGTGCACTGGATCTCGTT |
| *HIF1α* Forward | ATCCATGTGACCATGAGGAAATG |
| *HIF1α* Reverse | TCGGCTAGTTAGGGTACACTTC |
| *GLUT1* Forward | CATCCCATGGTTCATCGTGGCTGAACT |
| *GLUT1* Reverse | GAAGTAGGTGAAGATGAAGAACAGAAC |
| *HK2* Forward | GCCATCCTGCAACACTTAGGGCTTGAG |
| *HK2* Reverse | GTGAGGATGTAGCTTGTAGAGGGTCCC |
| *GPI* Forward | TATTGTGTTCACCAAGCTCACACC |
| *GPI* Reverse | TGGTAGAAGCGTCGTGAGAGGTC |
| *PFKL* Forward | GGAGAAGCTGCGCGAGGTTTAC |
| *PFKL* Reverse | ATTGTGCCAGCATCTTCAGCATGAG |
| *ALDOA* Forward | AGGCCATGCTTGCACTCAGAAGT |
| *ALDOA* Reverse | AGGGCCCAGGGCTTCAGCAGG |
| *GAPDH* Forward | TTCCGTGTCCCCACTGCCAACGT |
| *GAPDH* Reverse | CAAAGGTGGAGGAGTGGGTGTCGC |
| *PGK1* Forward | ATGTCGCTTTCTAACAAGCTGA |
| *PGK1* Reverse | GCGGAGGTTCTCCAGCA |
| *PGAM1* Forward | GGAAACGTGTACTGATTGCAGCCC |
| *PGAM1* Reverse | TTCCATGGCTTTGCGCACCGTCT |
| *ENO1* Forward | GACTTGGCTGGCAACTCTG |
| *ENO1* Reverse | GGTCATCGGGAGACTTGAA |
| *ENO2* Forward | TCATGGTGAGTCATCGCTCAGGAG |
| *ENO2* Reverse | ATGTCCGGCAAAGCGAGCTTCATC |
| *PKM2* Forward | GCCCGTGAGGCAGAGGCTGC |
| *PKM2* Reverse | TGGTGAGGACGATTATGGCCC |
| *LDHA* Forward | ATGGCAACTCTAAAGGATCA |
| *LDHA* Reverse | GCAACTTGCAGTTCGGGC |
